# Case Report: Achilles enthesitis as the presenting clue to occult sacroiliitis in hidradenitis suppurativa

**DOI:** 10.3389/fmed.2026.1932298

**Published:** 2026-08-19

**Authors:** Mohamed Bedaiwi

**Affiliations:** Rheumatology Unit, Department of Medicine, College of Medicine, King Saud University, Riyadh, Saudi Arabia

**Keywords:** adalimumab, axial spondyloarthritis, enthesitis, hidradenitis suppurativa, HLA-B27, sacroiliitis

## Abstract

Hidradenitis suppurativa is increasingly recognized as a systemic inflammatory disease with an established overlap with spondyloarthritis. Axial spondyloarthritis is usually suspected when patients report inflammatory back pain, which remains the entry feature of the ASAS axial spondyloarthritis classification criteria. We report a 31-year-old man with Hurley stage II hidradenitis suppurativa who presented with Achilles enthesitis supported by point-of-care ultrasound but denied any current or previous back pain lasting 3 months or longer, including inflammatory back pain and alternating buttock pain, prolonged morning stiffness, peripheral arthritis, dactylitis, uveitis, psoriasis, and gastrointestinal symptoms. During workup for asymptomatic microscopic hematuria, computed tomography of the pelvis incidentally showed bilateral sacroiliac joint abnormalities that prompted dedicated imaging. Magnetic resonance imaging of the sacroiliac joints showed findings meeting the consensus definition of active sacroiliitis, with bilateral subchondral bone marrow edema and early structural changes. The patient was human leukocyte antigen B27 positive, had elevated inflammatory markers, and had a first-degree family history of ankylosing spondylitis. Although the ASAS axial spondyloarthritis classification criteria were not fulfilled because the required history of back pain lasting 3 months or longer was absent, the patient fulfilled the ASAS peripheral spondyloarthritis classification criteria based on current Achilles enthesitis together with HLA-B27 positivity and sacroiliitis on imaging. Converging clinical, imaging, immunogenetic, and familial features supported a clinical diagnosis of spondyloarthritis associated with hidradenitis suppurativa. Adalimumab was started using the licensed hidradenitis suppurativa regimen. At 6 months, calcaneal tenderness and skin lesions had improved, inflammatory markers had normalized, and follow-up imaging showed substantial reduction in bone marrow edema. This case suggests that axial inflammation in hidradenitis suppurativa may be under-recognized when assessment relies exclusively on patient-reported back pain. Sacroiliac MRI should not be used routinely in all patients with hidradenitis suppurativa and enthesitis-like symptoms but may be considered after rheumatologic assessment when multiple clinical, laboratory, familial, or incidental imaging features collectively raise suspicion of spondyloarthritis.

## Introduction

Hidradenitis suppurativa (HS) is a chronic inflammatory skin disease characterized by recurrent painful nodules, abscesses, sinus tracts, and scarring in intertriginous areas (1). Beyond cutaneous involvement, HS shares inflammatory pathways with several immune-mediated conditions, including metabolic syndrome, inflammatory bowel disease, and spondyloarthritis (SpA), with overlapping roles for tumor necrosis factor (TNF), interleukin-17, and interleukin-23 signaling (2, 3). A measurable prevalence of SpA features among patients with HS has been reported (2, 4), and the relationship appears to be bidirectional, with HS symptoms also more frequent in axial SpA cohorts than expected (5). A subsequent systematic review and meta-analysis quantified the population-level association, showing significantly increased risks of inflammatory arthritis, spondyloarthritis, and ankylosing spondylitis among patients with HS compared with controls (6).

In current practice, axial SpA is included in the differential diagnosis when patients describe inflammatory back pain, which serves as the entry feature of the Assessment of SpondyloArthritis international Society (ASAS) classification criteria (7, 8). However, classification criteria are designed for research standardization rather than for individual diagnosis, and may exclude atypical presentations. Enthesitis is a recognized SpA manifestation and may be the dominant musculoskeletal complaint when axial symptoms are absent or mild.

We describe a young man with long-standing HS in whom active bilateral sacroiliitis was identified after an incidental computed tomography (CT) pelvis finding in the absence of any chronic back pain. The presenting musculoskeletal feature was ultrasound-supported Achilles enthesitis. This case is presented because sacroiliitis discovered incidentally during non-musculoskeletal imaging has rarely been emphasized as a diagnostic route in HS-associated SpA, and because the response to adalimumab using the HS regimen illustrates the practical advantage of recognizing the overlap before treatment initiation.

## Case description

A 31-year-old man with a 10-year history of HS was referred to the rheumatology department after developing posterior heel pain associated with calcaneal tenderness and elevated inflammatory markers. He explicitly denied any current or previous back pain lasting 3 months or longer, including inflammatory low back pain, alternating buttock pain, and prolonged morning stiffness; he also denied peripheral joint swelling, dactylitis, uveitis, psoriasis, or gastrointestinal symptoms. The patient had no personal history of inflammatory bowel disease. His father had ankylosing spondylitis, reportedly under rheumatology care and receiving biologic therapy, and his sister had psoriasis.

The patient was a non-smoker with a body mass index within the normal range. There was no documented history of diabetes, dyslipidemia, or other metabolic comorbidities. He did not report recent heel trauma, a change in footwear, or excessive mechanical loading prior to the onset of heel pain. Prior HS therapies included topical and oral treatments with incomplete control of cutaneous disease; detailed information about specific agents and durations was not available in the records reviewed.

His HS involved the axillae and groin and was classified as Hurley stage II, with recurrent nodules, sinus tracts, and scarring without extensive interconnected tracts. On examination, active HS lesions were observed in both axillae and the groin. Musculoskeletal examination revealed tenderness localized to the calcaneal insertion of the Achilles tendon bilaterally, more marked on the right, without swelling of the tendon body. No peripheral synovitis or dactylitis was observed. Nail examination findings were normal. The results of the modified Schober test, lateral lumbar flexion, and chest expansion were normal. Point-of-care musculoskeletal ultrasound of the symptomatic Achilles insertions was performed during the initial clinical assessment and showed findings considered consistent with insertional Achilles enthesitis. The ultrasound images were not archived and were therefore unavailable for inclusion or independent retrospective review; power Doppler findings could not be verified retrospectively.

Laboratory investigations showed a C-reactive protein (CRP) level of 34 mg/L (reference < 5 mg/L) and an erythrocyte sedimentation rate (ESR) of 58 mm/h (reference < 20 mm/h). Human leukocyte antigen B27 (HLA-B27) was positive. The results of complete blood count, renal and liver function tests, rheumatoid factor, and anti-cyclic citrullinated peptide antibodies were normal or negative. Tests for hepatitis B, hepatitis C, human immunodeficiency virus, and an interferon-gamma release assay for tuberculosis were negative.

**Figure 1:**
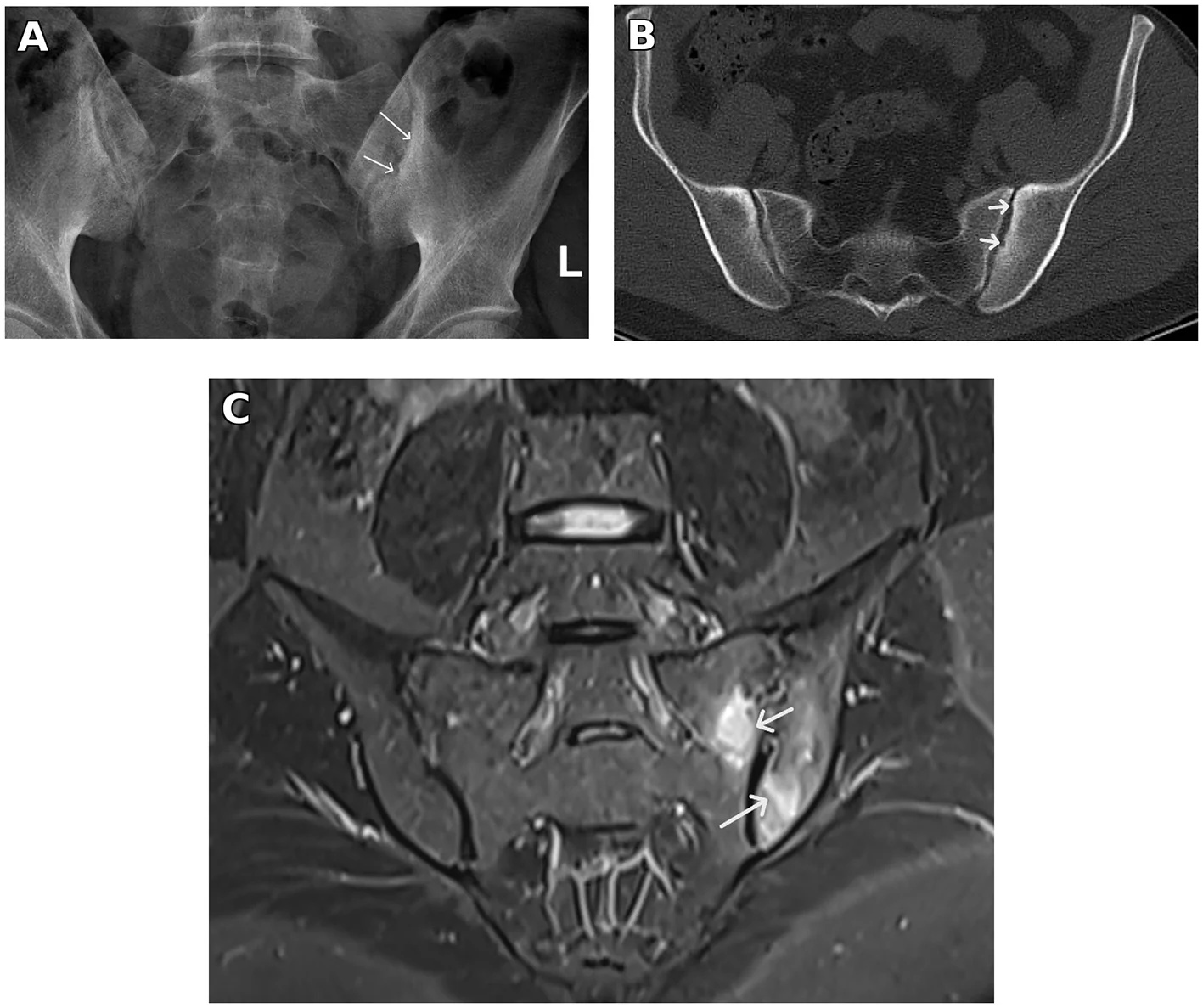
Baseline sacroiliac imaging. **(A)** Conventional sacroiliac-joint radiograph. White arrows indicate mild left-sided subchondral sclerosis and subtle articular-surface irregularity; the joint spaces remain preserved, without ankylosis, and definite radiographic sacroiliitis was not established. **(B)** Axial bone-window CT image. White arrows indicate subchondral sclerosis and focal articular-surface irregularity at the left sacroiliac joint, suggestive of possible early erosive change; the joint spaces remain preserved, without ankylosis. **(C)** Baseline semicoronal STIR MRI. White arrows indicate prominent subchondral bone marrow edema involving the iliac and sacral aspects of the left sacroiliac joint on the displayed slice. Additional inflammatory lesions were present bilaterally on adjacent slices, and the overall examination met the 2009 ASAS-OMERACT definition of active sacroiliitis on MRI.

An incidental finding occurred during a prior workup for asymptomatic microscopic hematuria. CT of the pelvis, performed as part of the evaluation, showed bilateral subchondral sclerosis and sacroiliac articular-surface irregularity, more conspicuous on the left, with focal irregularity suggestive of possible early erosive change, preserved joint spaces, and no ankylosis (Figure 1B). These findings were considered indeterminate and prompted dedicated assessment. Subsequent urological evaluation, including renal and bladder ultrasonography, cystoscopy, and urine cytology, was unremarkable; no urinary tract source of microscopic hematuria was identified. Conventional radiographs of the sacroiliac joints and whole spine were also obtained. The whole-spine radiograph was unremarkable. The sacroiliac-joint radiograph showed mild left-sided subchondral sclerosis and subtle articular-surface irregularity, with preserved joint spaces and no ankylosis; definite radiographic sacroiliitis was not established (Figure 1A). Because the sacroiliac findings were unexpected and converged with HS, Achilles enthesitis, HLA-B27 positivity, elevated inflammatory markers, and a first-degree family history of ankylosing spondylitis, dedicated magnetic resonance imaging (MRI) of the sacroiliac joints was performed.

MRI was performed using semicoronal short tau inversion recovery (STIR) and T1-weighted sequences and was reviewed by a radiologist with expertise in musculoskeletal imaging. STIR images demonstrated multiple foci of subchondral bone marrow edema on both the iliac and sacral sides of the joints bilaterally. The 2009 Assessment of SpondyloArthritis international Society-Outcome Measures in Rheumatology (ASAS-OMERACT) definition of active sacroiliitis on MRI requires at least one bone marrow edema lesion to be present on at least two consecutive slices or at least two lesions on a single slice (9). The 2016 ASAS-MRI working group update reaffirmed bone marrow edema as the defining lesion of active sacroiliitis, with structural lesions supporting the interpretation but not required (10). The imaging findings met this definition in this case (Figure 1C). T1-weighted images showed corresponding subchondral erosions and small areas of fat metaplasia, with no definite ankylosis. The structural changes seen on MRI were considered consistent with sacroiliitis.

The ASAS axial SpA classification criteria were not formally fulfilled because back pain lasting at least 3 months, the required entry feature, was absent. However, the patient fulfilled the ASAS peripheral SpA classification criteria: current Achilles enthesitis represented the entry manifestation, while HLA-B27 positivity and sacroiliitis on imaging were qualifying SpA features (11). Classification through the peripheral pathway does not exclude axial involvement; rather, it reflects the applicable classification route in a patient presenting with a peripheral manifestation without back pain lasting at least 3 months. The clinical diagnosis of SpA associated with HS was supported by converging evidence, including MRI findings meeting the ASAS-OMERACT definition of active sacroiliitis, sacroiliac abnormalities on CT, HLA-B27 positivity, ultrasound-supported Achilles enthesitis, elevated CRP and ESR, first-degree family history of ankylosing spondylitis, and active HS. Differential considerations included infectious sacroiliitis (considered unlikely given symmetric bilateral disease, absence of systemic infectious features, and the immunogenetic context), osteitis condensans ilii (unlikely given male sex, bilateral inflammatory MRI findings, and erosive change), diffuse idiopathic skeletal hyperostosis (unlikely at age 31 with active inflammation), psoriatic arthritis with axial involvement (considered given the sister's psoriasis but felt less likely because the patient had no personal psoriasis, no nail changes, no dactylitis, and no peripheral synovitis), and synovitis, acne, pustulosis, hyperostosis, and osteitis (SAPHO) syndrome, which was considered but felt less likely because anterior chest wall involvement, multifocal osteitis, hyperostosis, and palmoplantar pustulosis were absent, and the predominant cutaneous phenotype was typical HS.

Adalimumab was started using the licensed HS regimen: 160 mg subcutaneously at week 0, 80 mg at week 2, and 40 mg weekly from week 4 onwards (12). This regimen was chosen because adalimumab is effective in HS and non-radiographic axial SpA (12, 13), allowing a single biologic to address both disease domains.

**Figure 2:**
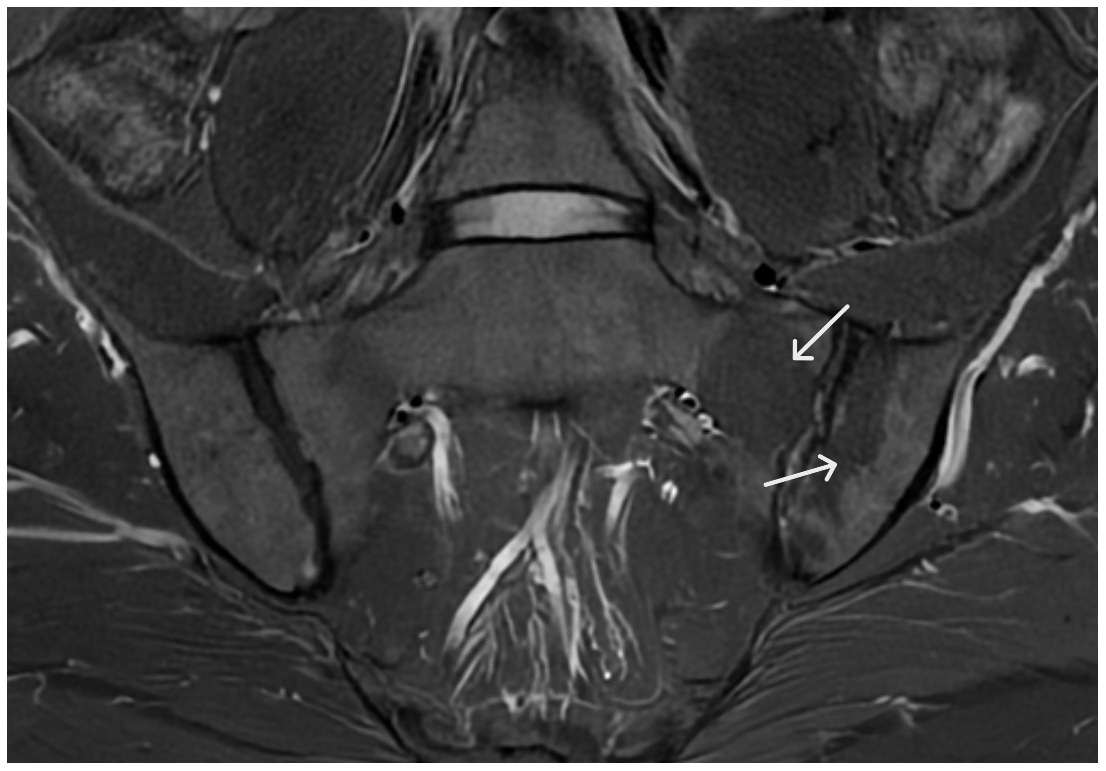
Follow-up semicoronal short tau inversion recovery (STIR) MRI of the sacroiliac joints obtained after 6 months of adalimumab therapy and in the same plane as the baseline study. White arrows indicate the corresponding anatomical sites of the previously demonstrated inflammatory lesions. Compared with baseline, there is marked interval reduction in subchondral bone marrow edema, with no definite residual focal fluid-sensitive sacroiliac signal abnormality on the displayed sequence. Bone marrow edema was not formally scored using the SPARCC system, and the comparison with baseline is therefore qualitative.

At 6-month follow-up, calcaneal tenderness had improved without complete resolution, HS lesions had improved with fewer active nodules and reduced drainage, and inflammatory markers had normalized (CRP 3 mg/L; ESR 14 mm/h). Repeat sacroiliac MRI showed substantial reduction in the previously observed subchondral bone marrow edema, with no definite residual focal fluid-sensitive signal abnormality on the available follow-up sequences, consistent with radiological improvement (Figure 2). Baseline and follow-up imaging were obtained in the same semicoronal plane, although bone marrow edema was not formally scored using the Spondyloarthritis Research Consortium of Canada (SPARCC) system. No adverse events were reported during the 6-month follow-up, and the patient remained on weekly adalimumab. The clinical, laboratory, and imaging course is summarized in Table 1.

**Table 1:** Timeline and clinical, laboratory, and imaging course.

Parameter	Finding
Age/sex	31 years/male
Hidradenitis suppurativa history	10 years; axillae and groin; Hurley stage II
Baseline characteristics	Non-smoker; body mass index within normal range; no documented diabetes, dyslipidemia, or metabolic syndrome; no recent heel trauma, change in footwear, or excessive mechanical loading reported
Prior HS treatments	Topical and oral therapies with incomplete control of cutaneous disease; details not available in the records reviewed
Presenting musculoskeletal feature	Bilateral calcaneal tenderness with point-of-care ultrasound findings consistent with insertional Achilles enthesitis; ultrasound images were not archived
Back pain	No current or previous back pain lasting 3 months or longer; inflammatory back pain features absent
Incidental imaging trigger	CT pelvis performed during evaluation of asymptomatic microscopic hematuria; subsequent urological evaluation including renal and bladder ultrasonography, cystoscopy, and urine cytology was unremarkable
Family history	Ankylosing spondylitis (father, under rheumatology care and on biologic); psoriasis (sister)
HLA-B27	Positive
CRP, baseline → 6 months	34 → 3 mg/L (reference < 5 mg/L)
ESR, baseline → 6 months	58 → 14 mm/h (reference < 20 mm/h)
CT pelvis	Bilateral subchondral sclerosis and sacroiliac articular-surface irregularity, more conspicuous on the left, with possible early erosive change; preserved joint spaces and no ankylosis; findings considered indeterminate and prompting dedicated MRI
Conventional radiography	Whole-spine radiograph unremarkable; sacroiliac-joint radiograph with mild left-sided subchondral sclerosis and subtle articular-surface irregularity, preserved joint spaces, and no ankylosis; definite radiographic sacroiliitis not established
MRI sacroiliac joints (baseline)	Bilateral subchondral bone marrow edema on consecutive slices, meeting the 2009 ASAS-OMERACT definition of active sacroiliitis on MRI; subchondral erosions and fat metaplasia on T1; no definite ankylosis
ASAS axial SpA criteria	Not formally fulfilled because back pain lasting at least 3 months, the required entry feature, was absent
ASAS peripheral SpA criteria	Fulfilled based on current Achilles enthesitis as the entry manifestation, together with HLA-B27 positivity and sacroiliitis on imaging
Clinical diagnosis	Spondyloarthritis associated with HS; ASAS peripheral SpA criteria fulfilled, with active sacroiliitis on MRI despite the absence of back pain lasting at least 3 months required for the axial SpA criteria
Treatment	Adalimumab 160 mg week 0, 80 mg week 2, 40 mg weekly from week 4 (HS regimen)
Six-month outcome	Improvement in calcaneal symptoms and HS lesions; normalization of CRP and ESR; substantial reduction in MRI bone marrow edema; no adverse events reported during six-month follow-up

ASAS, assessment of spondyloarthritis international society; CRP, C-reactive protein; CT, computed tomography; ESR, erythrocyte sedimentation rate; HLA-B27, human leukocyte antigen B27; HS, hidradenitis suppurativa; MRI, magnetic resonance imaging; SpA, spondyloarthritis.

## Patient perspective

The patient reported that he had not connected his skin disease with the heel symptoms or incidental pelvic findings, and the absence of back pain had made the diagnosis unexpected. After 6 months of adalimumab, he described the parallel improvement in skin and heel symptoms as the most noticeable change in his daily comfort.

## Discussion

This case illustrates a diagnostic route for spondyloarthritis in patients with HS that does not begin with inflammatory back pain. The patient had MRI sacroiliitis meeting the ASAS-OMERACT definition, with early structural change; however, the axial disease was clinically silent at its anatomical site and was first identified on a CT pelvis performed for an unrelated indication. Ultrasound-supported Achilles enthesitis provided the clinical context that prompted dedicated MRI. Imaging-based HS cohorts have shown frequent sacroiliac abnormalities, and emerging systematic assessment suggests that subclinical axial and entheseal SpA features may occur even when musculoskeletal symptoms are limited or absent (14, 15). The present case is consistent with this pattern at the level of an individual diagnostic encounter, and its practical importance is that it falls outside referral pathways that depend on patient-reported back pain.

The reported associations between HS and SpA are summarized in Table 2 and are not reiterated here. Briefly, screening of HS cohorts has identified SpA features in a meaningful minority (2, 4); isolated overlap cases with classical inflammatory back pain have been reported with a response to adalimumab (16); and the reverse association, increased HS symptoms in axial SpA cohorts, has also been described (5). Recent evidence further supports this interpretation: a systematic review and meta-analysis demonstrated significantly increased risks of inflammatory arthritis, spondyloarthritis, and ankylosing spondylitis among patients with HS (6), and an emerging prospective cross-sectional study reported a substantial frequency of subclinical axial and entheseal SpA features in HS, including MRI-confirmed sacroiliitis, even when musculoskeletal symptoms were limited or absent (15). The present case adds a different clinical message at the level of an individual encounter: in some patients with HS, the axial component may be present and active without producing symptoms that would otherwise trigger evaluation.

**Table 2:** Selected studies of HS-associated musculoskeletal disease and the position of the present case.

Study	Population/direction	Key finding	Position relative to present case
Richette et al. (2)	HS cohort screened for SpA features	Peripheral arthritis, inflammatory back pain, and enthesitis reported in HS-SpA overlap	Establishes occurrence of SpA features in HS
Fauconier et al. (4)	HS cohort with structured SpA assessment	SpA identified in a substantial subset; axial disease prominent	Supports increased SpA risk and importance of axial assessment
Bosnić et al. (16)	Single overlap case	HS with classical HLA-B27-positive ankylosing spondylitis and inflammatory back pain; response to adalimumab	Demonstrates therapeutic overlap but with typical axial symptoms
Rondags et al. (5)	Axial SpA cohort (reverse direction)	HS symptoms more prevalent than expected in axial SpA patients	Supports bidirectional clinical overlap
Schneider-Burrus et al. (14)	HS cohort with pelvic MRI assessment	High frequency of back pain and imaging features of axial spondyloarthropathy in HS	Supports that imaging-detected axial involvement in HS may be under-recognized by symptoms alone
Almuhanna et al. (6)	Meta-analysis (7 studies; 200,361 HS patients vs 385,599 controls)	Increased risk of inflammatory arthritis (OR 3.44), spondyloarthritis (OR 2.10), and ankylosing spondylitis (OR 1.89) in HS	Quantifies the population-level association supporting the present case as a clinically meaningful overlap
Bakay and Karstarli Bakay (15)	Prospective cross-sectional study of 120 HS patients	Subclinical axial and peripheral SpA features frequently detected, including MRI-confirmed sacroiliitis	Supports structured SpA assessment and careful case finding in HS
Present case	HS patient with ultrasound-supported Achilles enthesitis and incidentally detected sacroiliitis	MRI findings meeting the ASAS-OMERACT definition of active sacroiliitis without back pain lasting at least 3 months; ASAS peripheral SpA criteria fulfilled; identified after CT pelvis for asymptomatic hematuria; response to adalimumab	Illustrates incidentally detected axial inflammation as a diagnostic route in HS and the relevance of the peripheral SpA classification pathway

Two features deserve special emphasis. First, classification criteria and clinical diagnosis are not equivalent. The ASAS axial SpA criteria require back pain lasting at least 3 months with onset before the age of 45 years (8); therefore, this patient could not fulfill the axial criteria despite active MRI sacroiliitis. However, because he presented with current Achilles enthesitis and had both HLA-B27 positivity and sacroiliitis on imaging, he fulfilled the ASAS peripheral SpA classification criteria (11). This peripheral classification pathway does not imply that axial inflammation was absent; instead, it captures a patient whose presenting manifestation was peripheral enthesitis and who had no back pain lasting at least 3 months. Clinical diagnosis rested on the convergence of imaging, HLA-B27 positivity, ultrasound-supported enthesitis, family history, inflammatory markers, and HS, together with the absence of a more plausible alternative. Second, clinically silent or under-recognized sacroiliac inflammation has also been reported in HS imaging cohorts (14, 15), supporting the concept that musculoskeletal history alone may miss axial disease. The present case is consistent with this pattern: silence at the anatomical site did not equate to biological inactivity, given the active MRI lesions, changes on CT, and elevated inflammatory markers at baseline.

Another implication concerns structural disease. Despite the absence of inflammatory back pain, baseline MRI in this patient showed structural sacroiliac changes on T1-weighted images, including subchondral erosions and small areas of fat metaplasia. This observation suggests that the absence of axial symptoms does not necessarily exclude demonstrable sacroiliac structural change. Although a single case cannot determine the risk or rate of progression, it supports the careful evaluation of patients with HS and SpA features, especially when enthesitis-like symptoms, HLA-B27 positivity, elevated inflammatory markers not fully attributable to skin disease, or a family history of SpA are present. The ASAS axial SpA classification criteria, which require back pain lasting at least 3 months as the entry feature, may not capture such patients, and this gap is clinically relevant for both dermatology and rheumatology services.

The therapeutic course is informative. Adalimumab is licensed for moderate-to-severe HS and is effective in non-radiographic axial SpA (12, 13). Choosing the HS regimen was appropriate because it covered both disease domains with a single agent and allowed treatment response to be monitored across skin activity, calcaneal symptoms, inflammatory markers, and follow-up MRI. The parallel improvement across these domains, including a substantial reduction in MRI bone marrow edema, supports a true inflammatory response rather than symptomatic fluctuation, although natural variation in edema cannot be entirely excluded from a single case.

Several caveats apply. Point-of-care ultrasound supported Achilles insertional enthesitis, but the images were not archived and could not be included or independently reassessed; power Doppler and structural entheseal findings could therefore not be verified retrospectively. MRI sacroiliac changes were assessed qualitatively, not using SPARCC scoring, so the comparison between the baseline and follow-up images (Figures 1, 2) is qualitative rather than formally quantified. Conventional radiography did not establish definite radiographic sacroiliitis, and the CT structural abnormalities were subtle and indeterminate. In addition, the cutaneous response was assessed clinically and was not quantified using a validated HS response instrument such as HiSCR or IHS4. CT pelvis was performed for an unrelated reason; therefore, the diagnostic sequence is not generalizable as a screening pathway. Follow-up was limited to 6 months, and the durability of the response is therefore not established. Finally, this is a single observation, and no inferences about prevalence, screening yield, or causation should be drawn from it.

The teaching point is narrow and does not support routine sacroiliac MRI screening in all patients with HS and enthesitis-like symptoms. Imaging decisions should be individualized after rheumatologic evaluation and based on cumulative evidence. Features that may collectively increase suspicion include clinically convincing or ultrasound-supported enthesitis, HLA-B27 positivity, inflammatory-marker elevation not adequately explained by skin activity, a first-degree family history of SpA, or incidental sacroiliac abnormalities on prior imaging. In this case, the convergence of several independent features, rather than Achilles tenderness alone, justified dedicated sacroiliac MRI. Conversely, incidental sacroiliac abnormalities should be interpreted in their full clinical context and neither dismissed nor over-read.

## Conclusion

Sacroiliitis in HS may be active despite the absence of axial symptoms. In this patient, Achilles enthesitis provided relevant musculoskeletal context for an incidental CT abnormality, while HLA-B27 positivity, elevated inflammatory markers, and a first-degree family history further increased suspicion. Dedicated MRI confirmed active bilateral sacroiliitis. Although the ASAS axial SpA criteria were not fulfilled because the required history of back pain lasting at least 3 months was absent, the patient fulfilled the ASAS peripheral SpA criteria. This observation does not support routine MRI screening of all patients with HS and enthesitis-like symptoms; rather, it supports careful rheumatologic assessment and selective imaging when multiple clinical, laboratory, familial, or incidental imaging features converge. Collaboration between dermatology, rheumatology, and radiology may facilitate appropriate patient selection while avoiding unnecessary MRI use.

## Data Availability

The original contributions presented in the study are included in the article/Supplementary material, further inquiries can be directed to the corresponding author.
