# Supplementary material for: Case Report: Achilles enthesitis as the presenting clue to occult sacroiliitis in hidradenitis suppurativa
Source: Front Med (Lausanne). 2026 Aug 19;13:1932298. doi: 10.3389/fmed.2026.1932298 (PMC13535286; doi:10.3389/fmed.2026.1932298)
Supplement: Supplementary file 1 [file Table_1.docx]

**CARE Checklist (2013) of information to include when writing a case report**

**Manuscript:** “Case Report: Achilles enthesitis as the presenting clue to occult sacroiliitis in hidradenitis suppurativa” (Bedaiwi M). Page references correspond to the line-numbered submission file.

| **Item** | **Topic** | **CARE checklist item description** | **Reported** | **Location in manuscript** |
| --- | --- | --- | --- | --- |
| 1 | **Title** | The words “case report” appear in the title, together with the phenomenon of greatest interest. | Yes | Title (p. 1) |
| 2 | **Key words** | Two to five key words that identify topics in this case report. | Yes | Keywords (p. 1) |
| 3 | **Abstract** | (a) Introduction – what is unique and why is it important; (b) main symptoms and/or clinical findings; (c) main diagnoses, interventions, and outcomes; (d) conclusion. | Yes | Abstract (p. 1) |
| 4 | **Introduction** | Brief background summary of this case referencing the relevant medical literature. | Yes | Introduction (p. 1–2) |
| 5 | **Patient information** | (a) De-identified demographic and other patient information; (b) main concerns and symptoms; (c) medical, family, and psychosocial history including relevant genetic information; (d) relevant past interventions and their outcomes. | Yes | Case Description (p. 2–3); Table 1 |
| 6 | **Clinical findings** | Relevant physical examination and other significant clinical findings. | Yes | Case Description (p. 2–3) |
| 7 | **Timeline** | Relevant data from this episode of care organized as a timeline (figure or table). | Yes | Table 1 (clinical, laboratory, and imaging course) |
| 8 | **Diagnostic assessment** | (a) Diagnostic methods; (b) diagnostic challenges; (c) diagnostic reasoning including differential diagnosis; (d) prognostic characteristics where applicable. | Yes | Case Description (p. 3–4); Figure 1 |
| 9 | **Therapeutic intervention** | (a) Types of intervention; (b) administration of intervention (dosage, strength, duration); (c) changes in intervention with explanations. | Yes | Case Description (p. 4) |
| 10 | **Follow-up and outcomes** | (a) Clinician- and patient-assessed outcomes; (b) important follow-up diagnostic and other test results; (c) intervention adherence and tolerability; (d) adverse and unanticipated events. | Yes | Case Description (p. 4); Figure 2; Table 1 |
| 11 | **Discussion** | (a) Strengths and limitations of the management of this case; (b) relevant medical literature; (c) rationale for conclusions; (d) main takeaway lessons. | Yes | Discussion (p. 4–5); Table 2 |
| 12 | **Patient perspective** | The patient shares their perspective on the treatment(s) received. | Yes | Patient Perspective (p. 4) |
| 13 | **Informed consent** | Did the patient give informed consent? Provide if requested. | Yes | Ethics Statement (p. 6) |

*Reference: Gagnier JJ, Kienle G, Altman DG, Moher D, Sox H, Riley D; CARE Group. The CARE guidelines: consensus-based clinical case reporting guideline development. BMJ Case Rep. (2013). doi: 10.1136/bcr-2013-201554*
